# Effect of Cytokine Concentrations on Long-term Neurological Outcomes in Fetal Pleural Effusion Managed with Thoracoamniotic Shunt

**DOI:** 10.31662/jmaj.2024-0227

**Published:** 2024-12-13

**Authors:** Kenji Imai, Sho Tano, Kazuya Fuma, Seiko Matsuo, Takafumi Ushida, Hiroaki Kajiyama, Tomomi Kotani

**Affiliations:** 1 Department of Obstetrics and Gynecology, Nagoya University Graduate School of Medicine, Nagoya, Japan

**Keywords:** Fetus, Fetal therapy, Proinflammatory cytokine, IL-6, Development

## Introduction

Fetal pleural effusion (FPE) is a serious condition characterized by fluid accumulation in the pleural cavity, which often leads to fetal hydrops and significant perinatal morbidity and mortality ^(1), (2)^. It can arise from isolated lymphatic malformations or can be secondary to chromosomal abnormalities and structural anomalies. Managing FPE, particularly when complicated by hydrops, is challenging because of the potential for rapid progression to life-threatening conditions.

The thoracoamniotic shunt (TAS) is a critical intervention for severe FPE, facilitating the continuous drainage of pleural fluid and reducing the risk of hydrops progression ^(3)^. In a previous study, we reported the short-term outcomes of children with FPE, including their prognosis at 1.5 years of age ^(4)^. The children involved in that study are now 6 years old, thereby providing an opportunity to assess their long-term outcomes. This study not only evaluates these long-term outcomes but also explores the relationship between cytokine levels in FPE and long-term child prognosis. By expanding on our earlier findings, we provide deeper insights into the long-term effects of TAS and the pathophysiology of FPE.

## Methods

### Study design and participants

**Figure 1.**
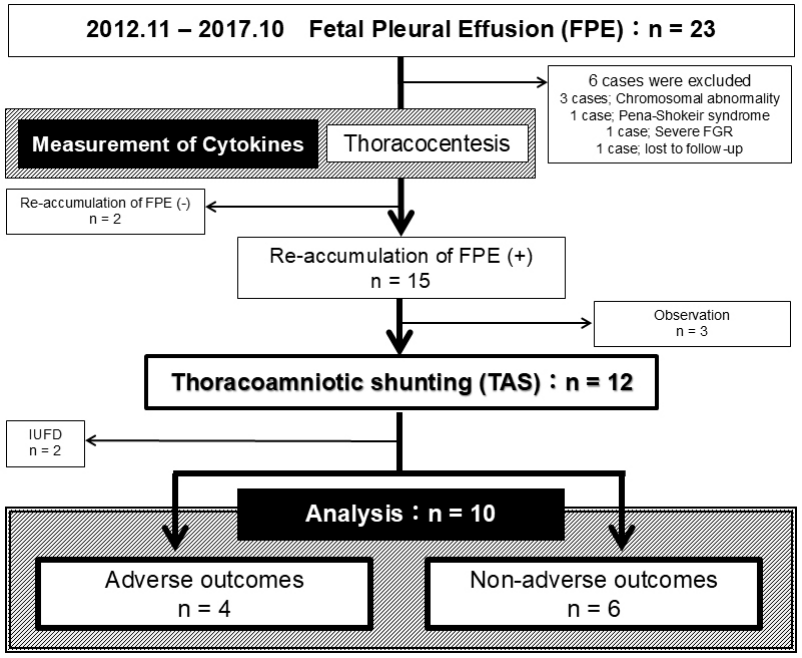
Flow diagram of study design. FGR, fetal growth restriction; FPE, fetal pleural effusion.

This retrospective cohort study examines the long-term neurological outcomes of 10 fetuses who underwent TAS for FPE at our tertiary care hospital between November 2012 and October 2017. All surviving patients are now at least 6 years old. All cases were free of chromosomal abnormalities and were classified as primary FPE. The selection process and criteria for these 10 cases are detailed in Figure 1. This study was approved by our hospital’s ethics committee (approval number 20180107). TAS was performed using a double-basket catheter under ultrasound guidance, following a standardized protocol ^(5)^. Cases of pleural fluid reaccumulation after TAS were managed with a second shunt if necessary.

Cytokine levels in FPE were analyzed using data previously published in our study ^(4)^. The multiplex immunoassay used in this study measured 40 cytokines in fetal pleural fluid samples.

Adverse long-term outcomes were defined as developmental disorders, such as low intelligence quotient (IQ), attention-deficit hyperactivity disorder (ADHD), and developmental delay. These outcomes were assessed by specialists at our hospital using the Kyoto Scale of Psychological Development and DSM-5 criteria.

### Statistical analysis

Data were analyzed using SPSS version 28. Continuous variables were reported as medians with interquartile ranges, and categorical variables were reported as frequencies and percentages. Comparative analyses were performed using the Mann-Whitney U test or chi-square test, as appropriate, with a significance level set at p < 0.05. Cytokine concentrations that showed significant differences were further examined using Spearman’s rank correlation.

## Results

Table 1 presents the background characteristics of patients categorized into adverse and non-adverse outcome groups. Although the gestational ages at delivery were nearly identical, infants in the adverse outcome group had clearly lower birth weights compared with those in the non-adverse outcome group, which may have contributed to poorer outcomes. Additionally, there was a higher incidence of fetal hydrops in the adverse outcome group, and these patients required more frequent postnatal interventions, such as thoracic drainage and octreotide or steroid therapy. However, none of these differences were statistically significant.

**Table 1.** Comparison of Characteristics with and without Adverse Outcomes.

	Adverse outcomes (*n* = 4)	Non-adverse outcomes (*n* = 6)	p
*Maternal characteristics*			
Maternal age (years)	31.0 (29.5-33.5)	33.5 (30.0-34.8)	0.668
Primiparity	2 (50.0)	5 (83.3)	0.500
Infertility treatment	1 (25.0)	1 (16.7)	1.00
Body mass index	21.5 (21.0-23.7)	19.8 (18.5-21.6)	0.088
GA at birth (weeks)	36.0 (35.3-36.3)	35.5 (34.0-37.0)	0.912
Cesarean section	3 (75.0)	5 (83.3)	1.00
*Fetal/Placental characteristics*			
GA at initial thoracocentesis (weeks)	30.5 (27.8-32.0)	27.0 (24.5-29.5)	0.454
Bilateral FPE	3 (75.0)	4 (66.7)	1.00
Large amount of FPE	3 (75.0)	4 (66.7)	1.00
Total cell counts in FPE (/μL)	871.7 (694.2-3090)	5100 (1259-13090)	1.00
Percentage of lymphocytes in FPE (%)	95.2 (92.2-97.6)	96.3 (88.6-98.5)	1.00
Total protein in FPE (g/L)	18.7 (16.1-21.9)	22.1 (15.8-24.8)	0.831
Hydrops	3 (75.0)	2 (33.3)	0.524
Polyhydramnios	1 (25.0)	4 (66.7)	0.524
Histological chorioamnionitis	1 (25.0)	1 (16.7)	1.00
*Neonatal characteristics*			
Birth weight* (g)	2415 (2276-2546)	2854 (2587-2904)	0.201
Male	2 (50.0)	5 (83.3)	0.500
5-min Apgar score <7*	2 (50.0)	2 (33.3)	1.00
Umbilical cord pH*	7.35 (7.33-7.37)	7.34 (7.30-7.38)	1.00
Umbilical cord BE*	−1.6 (−2.1-−0.1)	0.0 (−2.2-1.3)	1.00
Intubation*	3 (75.0)	5 (83.3)	1.00
Thoracic drainage*	3 (75.0)	1 (16.7)	1.00
Octreotide therapy*	3 (75.0)	3 (50.0)	0.571
Steroid therapy*	2 (50.0)	2 (33.3)	1.00

Data are presented as median (interquartile ranges) or n (%). *The cases of intrauterine death were excluded from the analysis. GA, gestational age; FPE, fetal pleural effusion; pH, potential of hydrogen; BE, base excess.

Table 2 details the information on the 10 cases included in the study, such as follow-up duration and the presence of fetal hydrops. The median follow-up period for survivors was 90.9 (72-120) months. Four of these cases were classified as having adverse outcomes, including two cases of low IQ, one case of ADHD, and one case of delayed language development.

**Table 2.** Individual Characteristics and Long-Term Outcomes of FPE.

Patient no.	Follow-up (m)	GA at TC (w)	GA at TAS (w)	GA at birth (w)	Location of FPE	Fetal hydrops	Histological CAM	Indication for birth	Birth weight (g)	Gender	UmA pH	UmA BE	Long-term outcome
1	120	23	24	34	Bilateral	Yes	Negative	NRFS	2506	Male	7.29	−2.7	Typical development
2	103	30	30	32	Bilateral	No	Negative	HDP	2004	Female	7.40	1.5	Typical development
3	86	24	25	37	Bilateral	No	Positive	Full term	2828	Male	7.30	−6.1	Typical development
4	77	33	33	34	Bilateral	No	Negative	NRFS	2912	Male	7.42	1.8	Typical development
5	72	26	27	37	Unilateral	No	Negative	Full term	2880	Male	7.35	0.8	Typical development
6	72	28	30	37	Unilateral	No	Negative	Full term	3038	Male	7.31	1.0	Typical development
7	116	33	33	36	Unilateral	No	Positive	Labor onset	2568	Male	7.29	−2.2	Low IQ at age 6
8	93	21	25	37	Bilateral	Yes	Negative	Full term	2227	Female	7.35	−2.0	ADHD at age 7
9	90	30	30	33	Bilateral	Yes	Negative	Labor onset	2292	Male	7.35	−1.1	Developmental delay at age 7 (Language)
10	80	31	31	38	Bilateral	Yes	Positive	Full term	2538	Female	7.43	3.1	Low IQ at age 6

ADHD, attention-deficit hyperactivity disorder; BE, base excess; CAM, chorioamnionitis; FPE, fetal pleural effusion; GA, gestational age; HDP, hypertensive disorders of pregnancy; IQ, intelligence quotient; m, months; NRFS, non-reassuring fetal status; pH, potential of hydrogen; TAS, thoracoamniotic shunt; TC, thoracocentesis; UmA, umbilical artery; w, weeks.

**Figure 2.**
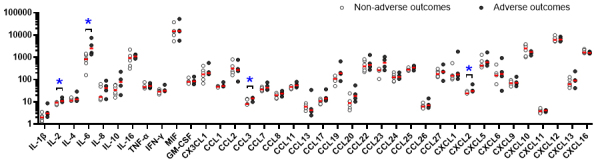
Logarithmic distribution of levels of 38 cytokines in fetal pleural effusion. Data are presented as a scatterplots with median values. CCL, chemokine ligand; IL, interleukin.

Among the 40 cytokines measured, the concentrations of chemokine ligand (CCL)15 and CCL21 were below the measurable limits in several cases; therefore, these two cytokines were excluded from further analysis. Out of the remaining 38 cytokines evaluated, interleukin (IL)-2, IL-6, the C-C motif CCL3, and CXCL2 were significantly elevated in the adverse outcome group (Supplemental Table 1). IL-6 was notably abundant in FPE, with marked differences in concentration between the two groups (Figure 2).

**Figure 3.**
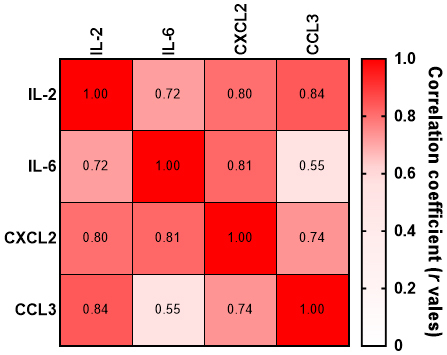
Correlation matrix heat map showing Spearman’s *r* value for the correlation between each cytokine pair. CCL, chemokine ligand; IL, interleukin.

Cytokines often operate in complex networks with overlapping roles in immune responses and inflammation. In our analysis, strong correlations were observed between IL-2, IL-6, CCL3, and CXCL2. Therefore, a potential collective role in the pathophysiology of FPE was identified for these cytokines (Figure 3).

Receiver operating characteristic analysis was performed for IL-2, IL-6, CCL3, and CXCL2. The results were as follows: IL-2 had an AUC of 0.985 (95% CI: 0.864-1.000), cutoff of 9.89 ng/mL, sensitivity of 83.3%, and specificity of 100%; IL-6 had an AUC of 0.958 (95% CI: 0.843-1.000), cutoff of 1346.4 pg/mL, sensitivity of 83.3%, and specificity of 100%; CCL3 had an AUC of 0.917 (95% CI: 0.723-1.000), cutoff of 14.46 pg/mL, sensitivity of 100%, and specificity of 75.0%; and CXCL2 had an AUC of 0.917 (95% CI: 0.728-1.000), cutoff of 28.98 ng/mL, sensitivity of 83.3%, and specificity of 100%.

## Discussion

This study is the first to thoroughly analyze cytokine concentrations specifically in patients with FPE who underwent TAS. By focusing on this particular group, our research provides valuable insights into factors that may predict long-term neurological outcomes after TAS. This focus is crucial because identifying prognostic markers in TAS-treated FPE can significantly improve patient care and contribute to a better understanding of the pathophysiology of FPE.

A key finding of this study was the elevated level of IL-6 in FPE. IL-6 was notably abundant, with concentrations significantly higher than those of other cytokines. The large difference between the two groups suggests that IL-6 plays a key role in the pathophysiology of FPE and is critical for determining prognosis, especially in patients who underwent TAS. This finding is consistent with the concept of fetal inflammatory response syndrome, which involves heightened inflammation *in utero* and is associated with poor neonatal outcomes ^(6), (7), (8)^. Studies have shown that high IL-6 levels in umbilical cord blood are correlated with adverse outcomes, and our study extends this observation to TAS-treated FPE cases ^(9)^. The elevated IL-6 levels suggest that this cytokine could be an important biomarker for predicting adverse long-term outcomes in this specific patient group. Additionally, studies on adult pleural effusion have demonstrated that cytokine profiles can predict disease outcomes, particularly in cases involving cancer and infections. These parallels suggest that cytokine analysis could also be useful for guiding treatment and predicting outcomes in TAS-treated FPE cases ^(10), (11)^.

Generally, the presence of fetal hydrops is recognized as a poor prognostic factor for fetal outcomes ^(1)^. In our cohort, the frequency of fetal hydrops was indeed higher in the adverse outcome group, although the small sample size may have prevented this difference from reaching statistical significance. Conversely, the fact that cytokine levels, such as IL-6, were significantly different even in this small cohort suggests that cytokine profiling may be a highly valuable prognostic tool in FPE cases.

Despite these findings, the reasons for elevated cytokine levels in TAS-treated FPE cases are not fully understood. Whether these elevations are due to infection (at least the incidence of chorioamnionitis did not significantly differ between the two groups), immune response, or tissue damage remains unclear. Further research is needed to investigate these underlying causes, which could lead to better management strategies for FPE in the context of TAS.

In conclusion, this study highlights the potential of cytokine profiling, especially IL-6, for predicting long-term outcomes in fetuses with FPE who undergo TAS. By focusing on this specific patient group, our research provides important insights that could guide future studies and improve clinical outcomes through targeted interventions.

## Article Information

### Conflicts of Interest

None

### Acknowledgement

We thank Sachiko Morisaki for her valuable technical support and Editage (www.editage.jp) for editing the English language.

### Author Contributions

Kenji Imai contributed to the concept and design of the study, performed the statistical analyses, and drafted the first version of the manuscript. Sho Tano and Kazuya Fuma were involved in data collection and management. Seiko Matsuo and Takafumi Ushida assisted in the data analysis and interpretation. Hiroaki Kajiyama and Tomomi Kotani provided critical feedback and oversight throughout the study and manuscript preparation, ensuring the scientific rigor and integrity of the research. All authors contributed to data interpretation, critically reviewed the manuscript, and approved the final version of the manuscript.

### Approval by Institutional Review Board (IRB)

Written informed consent was obtained from all participants. The Institutional Review Board of Nagoya University approved this study (approval number: 20180107).

## Supplement

Supplemental Table 1
