# Supplementary material for: Effect of Cytokine Concentrations on Long-term Neurological Outcomes in Fetal Pleural Effusion Managed with Thoracoamniotic Shunt
Source: JMA J. 2024 Dec 13;8(1):288–92. doi: 10.31662/jmaj.2024-0227 (PMC11799429; doi:10.31662/jmaj.2024-0227)
Supplement: Supplemental Table 1 [file 2433-3298-8-1-0288-s001.pdf]

Supplemental table 1. Cytokine levels in fetal pleural effusion

	Adverse outcomes (n=4)	Non-adverse outcomes (*n*=6)	*p*
IL-1β (pg/mL)	3.04 (2.66 to 4.58)	2.00 (1.52 to 2.24)	0.136
**IL-2 (pg/mL)**	**10.85 (9.89 to 12.81)**	**9.23 (8.32 to 9.69)**	**0.018**
IL-4 (pg/mL)	12.87 (11.57 to 17.82)	11.24 (11.07 to 13.35)	0.330
**IL-6 (pg/mL)**	**2530.5 (1535.7 to 4461.0)**	**811.7 (601.6 to 1163.7)**	**0.019**
IL-8 (pg/mL)	41.90 (27.78 to 60.70)	16.31 (14.33 to 40.56)	0.394
IL-10 (pg/mL)	77.46 (45.04 to 132.5)	34.15 (22.53 to 51.55)	0.286
IL-16 (pg/mL)	1101.2 (927.8 to 1277.2)	941.9 (489.0 to 1173.5)	0.394
TNF-α (pg/mL)	51.17 (45.81 to 58.73)	46.79 (46.16 to 49.59)	0.522
IFN-γ (pg/mL)	31.53 (30.17 to 39.35)	28.80 (25.57 to 31.14)	0.134
MIF (pg/mL)	15218 (12406 to 24989)	14755 (11086 to 14985)	0.522
GM-CSF (pg/mL)	83.75 (75.68 to 99.41)	78.91 (74.93 to 81.01)	0.522
CX3CL1 (pg/mL)	197.0 (178.7 to 300.9)	172.5 (113.9 to 239.3)	0.394
CCL1 (pg/mL)	51.98 (50.81 to 58.84)	47.35 (45.38 to 49.13)	0.055
CCL2 (pg/mL)	271.9 (203.2 to 418.8)	316.86 (210.1 to 411.7)	0.831
**CCL3 (pg/mL)**	**14.53 (13.33 to 14.78)**	**8.09 (7.73 to 11.75)**	**0.033**
CCL7 (pg/mL)	43.70 (35.82 to 57.11)	35.72 (33.30 to 40.33)	0.201
CCL8 (pg/mL)	23.86 (21.80 to 26.10)	19.98 (15.98 to 21.12)	0.201
CCL11 (pg/mL)	55.66 (49.69 to 64.94)	46.09 (40.65 to 50.60)	0.055
CCL13 (pg/mL)	4.22 (3.40 to 12.12)	6.12 (5.30 to 8.25)	0.286
CCL17 (pg/mL)	14.12 (12.91 to 20.32)	10.91 (8.56 to 11.86)	0.088
CCL19 (pg/mL)	191.5 (158.7 to 315.2)	108.0 (91.58 to 140.8)	0.136
CCL20 (pg/mL)	18.19 (13.42 to 30.18)	8.87 (5.76 to 10.76)	0.136
CCL22 (pg/mL)	455.4 (355.0 to 716.3)	396.7 (274.0 to 635.7)	0.522
CCL23 (pg/mL)	570.6 (352.0 to 834.9)	267.5 (247.4 to 285.9)	0.055
CCL24 (pg/mL)	131.0 (111.7 to 163.5)	127.2 (95.48 to 153.3)	0.831
CCL25 (pg/mL)	347.8 (312.8 to 378.5)	280.9 (259.7 to 291.2)	0.136
CCL26 (pg/mL)	6.94 (6.23 to 9.11)	6.19 (5.47 to 6.67)	0.286
CCL27 (pg/mL)	224.1 (187.2 to 290.4)	177.9 (141.4 to 228.9)	0.670
CXCL1 (pg/mL)	168.8 (139.6 to 591.7)	147.1 (122.9 to 152.7)	0.522
**CXCL2 (pg/mL)**	**30.50 (29.27 to 39.08)**	**23.96 (23.51 to 26.33)**	**0.032**
CXCL5 (pg/mL)	597.0 (518.6 to 902.0)	429.2 (375.3 to 616.1)	0.286
CXCL6 (pg/mL)	180.8 (114.8 to 396.0)	153.9 (84.12 to 184.8)	0.831
CXCL9 (pg/mL)	71.17 (63.10 to 89.80)	69.83 (62.75 to 77.83)	0.831
CXCL10 (pg/mL)	1730.3 (1484.7 to 1980.8)	2509.7 (1360.6 to 3377.7)	0.394
CXCL11 (pg/mL)	4.16 (3.90 to 4.32)	3.82 (3.32 to 4.42)	0.831
CXCL12 (pg/mL)	6266.1 (5428.5 to 7387.7)	6091.4 (5289.5 to 7089.5)	0.831

CXCL13 (pg/mL)	91.32 (77.23 to 129.2)	62.08 (53.40 to 89.52)	0.670
CXCL16 (pg/mL)	1574.4 (1503.4 to 1680.6)	1664.0 (1617.3 to 2017.9)	0.286

Data are presented as medians (interquartile ranges). IL, Interleukin; TNF-α, Tumor necrosis factor-α; IFN-γ, Interferon-γ; MIF, Macrophage migration inhibitory factor; GM-CSF, Granulocyte monocyte-colony stimulating factor; CX3CL1, Chemokine CX3C motif ligand 1; CCL, Chemokine C-C motif ligand; CXCL, Chemokine C-X-C motif ligand.
